# Is acromioplasty necessary in the setting of full-thickness rotator cuff tears? A systematic review

**DOI:** 10.1007/s10195-015-0353-z

**Published:** 2015-05-24

**Authors:** Filippo Familiari, Alan Gonzalez-Zapata, Bruno Iannò, Olimpio Galasso, Giorgio Gasparini, Edward G. McFarland

**Affiliations:** Division of Shoulder Surgery, Department of Orthopaedic Surgery, The Johns Hopkins University, 10753 Falls Road, Pavilion II, Suite 215, Lutherville, MD 21093 USA; Department of Orthopaedic and Trauma Surgery, Magna Græcia University, Catanzaro, Italy

**Keywords:** Acromioplasty, Surgery, Rotator cuff tear, Subacromial decompression, Coracoacromial ligament, Systematic review

## Abstract

**Background:**

The benefits of acromioplasty in treating rotator cuff disease have been debated. We systematically reviewed the literature regarding whether acromioplasty with concomitant coracoacromial(CA) release is necessary for the successful treatment of full-thickness rotator cuff tears.

**Materials and methods:**

We identified randomized controlled trials that reported on patients who underwent rotator cuff repair with or without acromioplasty and used descriptive statistics to summarize the findings.

**Results:**

Four studies fulfilled the inclusion criteria. They reported on 354 patients (mean age, 59 years; range 3–81 years) with a mean follow-up of 22 months (range 12–24 months). There were two level-I and two level-II studies. Two studies compared rotator cuff repair with versus without acromioplasty, and two studies compared rotator cuff repair with versus without subacromial decompression (acromioplasty, CA ligament resection, and bursectomy). The procedures were performed arthroscopically, and the CA ligament was released in all four studies. There were no statistically significant differences in clinical outcomes between patients treated with acromioplasty compared with those treated without acromioplasty.

**Conclusions:**

This systematic review of the literature does not support the routine use of partial acromioplasty or CA ligament release in the surgical treatment of rotator cuff disease. In some instances, partial acromioplasty and release of the CA ligament can result in anterior escape and worsening symptoms. Further research is needed to determine the optimum method for the operative treatment of full-thickness rotator cuff tears.

**Level of evidence:**

Level I, systematic review of level I and II studies.

## Introduction

Shoulder pain has been described as the second-most common musculoskeletal disorder after low back pain [1–4]. Disorders of the rotator cuff, commonly called “impingement,” have been reported to be the leading cause of pain in the shoulder [5, 6]. In 1949, Armstrong [7] first suggested that compression of the bursa and rotator cuff tendons under the acromion causes supraspinatus syndrome. Subsequently, Neer [8] stated that 95 % of rotator cuff tears were caused by mechanical impingement and reported successful treatment with partial anterior acromioplasty. Later, the same author described three stages in the development of impingement: stage I, involving edema and hemorrhage; stage II, an irreversible stage involving tendinitis and fibrosis; and stage III, involving severe tendon degeneration and tearing [9]. A subsequent study using conventional radiographs reported a relationship between the shape of the acromion [flat (type I), curved (type II), or hooked (type III)] and the presence of rotator cuff disease [10]. Although these studies confirmed an association between rotator cuff disease and acromial shape, a causal relationship between the shape of the acromion and rotator cuff disease was not established [11, 12].

The procedure of reshaping the acromion with a partial acromioplasty to relieve mechanical pressure on the rotator cuff was widely adopted in open rotator cuff repair. The ability to perform an arthroscopic partial acromioplasty was first described by Ellman [13] in 1987. The risks and benefits of open acromioplasty compared with the arthroscopic approach have been identified in a series of studies, as summarized by Spangehl et al. [14]. The major advantage of the open procedure was that it was technically easier to perform and required less surgeon expertise [14]. The advantages of the arthroscopic approach theoretically included improved cosmetic appearance of the surgical scar, preservation of the deltoid muscle, and faster recovery [14].

Subsequent studies questioned the role of the acromion in the production of rotator cuff disease [15, 16]. Tibone et al. [17] found that partial acromioplasty did not result in improvement of pain in athletic individuals with “impingement.” Published reviews of the efficacy of partial acromioplasty for rotator cuff symptoms found that the results were not as good as expected, with failure rates of 15–20 % [18, 19].

In 2001, Goldberg et al. [20] reported the first clinical study to suggest that acromioplasty for full-thickness rotator cuff tears was not necessary for a successful surgical result; this was subsequently confirmed by McAllister et al. [21]. Both studies reported on full-thickness rotator cuff repairs performed without acromioplasty, thus preserving the integrity of the coracoacromial (CA) arch and the deltoid insertion. They found statistically significant improvements in all clinical outcomes evaluated and advocated abandonment of partial acromioplasty and CA ligament release for the treatment of rotator cuff disease [20, 21].

These studies led to a reassessment not only of the role of the acromion in the development of rotator cuff disease but also of the concept of “impingement” itself [22, 23]. Most of these studies suggest that rotator cuff disease is a multifactorial process of both intrinsic causes (rotator cuff degeneration with age) and extrinsic causes (contact with other structures, high tensile load) [24, 25]. However, subsequent clinical studies have indicated that the role of partial acromioplasty and CA ligament release in the surgical treatment of rotator cuff disease should be reassessed. It has been shown that acromioplasty with CA ligament release may lead to increases in anterosuperior and superior glenohumeral instability [26–28].

The purpose of this review was to systematically evaluate published clinical studies as they relate to the need for partial acromioplasty with concomitant release of the CA ligament in the treatment of full-thickness rotator cuff tears.

## Materials and methods

Three independent reviewers (F.F., A.G.Z., and E.G.M.) performed a review of the literature using the MEDLINE/PubMed, Excerpta Medica/EMBASE, and Cochrane Register of Controlled Trials databases. Our purpose was to identify and include all English-language randomized controlled trials (level I or II) on the role of acromioplasty with concomitant release of the CA ligament in the treatment of full-thickness rotator cuff tears. We searched using the keywords “acromioplasty,” “arthroscopic acromioplasty,” “open acromioplasty,” “subacromial decompression,” and “coracoacromial ligament” (“Appendix”). Only prospective, randomized studies that reported on patients who underwent rotator cuff repair with or without acromioplasty were included.

**Fig. 1:**
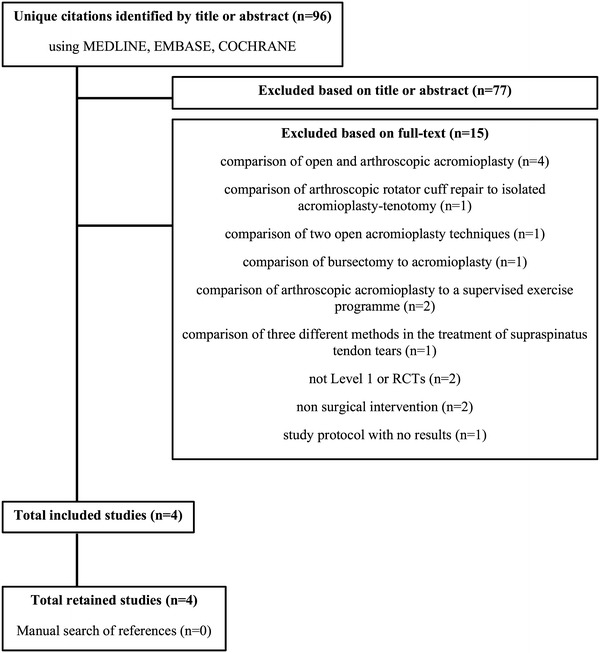
Flowchart for the literature search

Our search identified 96 pertinent abstracts or full-text articles. Reference sections of all accessed papers were searched for any undetected studies. These articles were reviewed and cross-referenced to exclude repeated references. Nineteen of these were considered relevant, and the full text of each was reviewed to determine eligibility. Seventy-seven articles were excluded on the basis of titles or abstracts, and 15 were excluded on the basis of full-text review. Biomechanical reports, animal and cadaver studies, in vitro studies, case reports, literature reviews, technical notes, letters to the editor, instructional courses, studies comparing different techniques, study protocols with no results, and studies of nonsurgical interventions were excluded. The remaining four articles [29–32] met the inclusion criteria and were analyzed in this systematic review (Fig. 1).

This review includes only articles that meet accepted quality standards for design and reporting as described by Wright et al. [33] and Spindler et al. [34] and according to the CONSORT statement [35].

For studies that used similar outcome measures, we pooled the results to generate a summary outcome—the frequency-weighted mean (calculated by weighting the mean value for each study by the number of patients in that study). If both preoperative and postoperative values for the outcome were available, we used the frequency-weighted means to calculate a *P* value for the change; a value of *P* < 0.05 was considered statistically significant.

We extracted the following data: study year, country, study design, and presence of control group; primary and secondary hypotheses; primary and secondary outcomes; basic study characteristics, including number of enrolled patients, patient age, patient sex, length of follow-up, and study group comparability at baseline; potential sources of bias; use of validated questionnaires; statistical methods and consultation with a biostatistician; presence of independent examiners; differences in rehabilitation protocols between groups; and results (Table 1). Data were extracted from each of the selected papers independently by two evaluators (F.F. and A.G.Z.). There was agreement regarding inclusion or exclusion in all cases. Specific data extracted included the degree of rotator cuff abnormality, the outcome measures (where available), preoperative versus postoperative range of motion, and patient satisfaction and pain relief.

**Table 1:** Details of included studies

Characteristics	Abrams et al. [29]	Gartsman and O’Connor [30]	MacDonald et al. [31]	Milano et al. [32]
Year	2014	2004	2011	2007
Country	United States	United States	Canada	Italy
Study design	RCT	RCT	RCT	RCT
Level of evidence	II	II	I	I
Procedures	ACR versus ACR-A	ACR versus ACR-SD	ACR versus ACR-A	ACR versus ACR-SD
Inclusion criteria	Full-thickness superior rotator cuff tear	Isolated, repairable full-thickness supraspinatus tendon tear and type 2 acromion	Full-thickness rotator cuff tear	Full-thickness rotator cuff tear and type 2 or 3 acromion
No. of patients	95	93	86	80
Mean age in years	58.8 (SD ±8.1)	59.7 (range 37–81)	56.8 (range 33–77)	60.3 (SD ±8.3)
Mean follow-up in months	24	15.6 (SD ±3.3)	24	24
Study outcome measures	ASES, SST, UCLA, VAS, Constant–Murley	ASES	ASES, ROM, WORC	Constant–Murley, DASH, Work-DASH
Study characteristics comparable at baseline	Yes	Yes	Yes	Yes
Use of validated questionnaires	Yes	Yes	Yes	Yes
Presence of independent examiners	Yes	No	Not reported	Yes
Difference in rehabilitation protocols in groups	No	No	Yes	No

*ACR* Arthroscopic cuff repair, *ACR-A* arthroscopic cuff repair with acromioplasty, *ACR-SD* arthroscopic cuff repair with subacromial decompression, *ASES* American Shoulder and Elbow Surgeons score, *DASH* Disabilities of the Arm, Shoulder, and Hand questionnaire, *RCT* randomized controlled trial, *ROM* range of motion, *SD* standard deviation, *SST* Simple Shoulder Test, *UCLA* University of California–Los Angeles score, *VAS* Visual Analog Scale for pain, *WORC* Western Ontario Rotator Cuff Index, *Work-DASH* Work-Disabilities of the Arm, Shoulder, and Hand questionnaire

## Results

There were two level-I [31, 32] and two level-II studies [29, 30] that met the inclusion criteria. These four studies reported on a total of 354 patients (range 80–95 per study) [29–32]. The mean patient age was 59 years (range 3–81). Three studies indicated patients’ sex, with 159 (63 %) males and 93 (37 %) females [29, 31, 32]. Two studies compared rotator cuff repair with and without acromioplasty [29, 31], and two studies compared rotator cuff repair with and without subacromial decompression (acromioplasty, CA ligament resection, and bursectomy) [30, 32]. The procedures were performed arthroscopically, and the CA ligament was released in all four studies [29–32]. Patients were followed for a mean of 22 months (range 12–24 months).

The outcomes included pain relief [29–31], range of motion [29, 31], and patient- and disease-specific outcome measures (disease-specific quality of life, shoulder-specific outcome measures) [29–32] at final follow-up (Table 2). None of the studies evaluated postoperative patient satisfaction or rotator cuff integrity. There were no statistically significant differences in clinical results between patients treated with acromioplasty versus those treated without acromioplasty in all studies [29–32]. The variability in functional outcome measures reported across trials made a pooled analysis possible for only American Shoulder and Elbow Surgeons scores [29–31] and Constant–Murley scores [29, 32], and no statistically significant differences were found (*P* = 0.938 and *P* = 0.673, respectively) (Table 3). None of the studies measured patient satisfaction or outcomes in a nonparametric manner such as poor, fair, good, or excellent.

**Table 2:** Postoperative results of validated questionnaires

Study	Procedure	ASES	WORC	UCLA	CM	VAS	SST	DASH	Work-DASH
MacDonald et al. [31]	ACR	85.6	80.7						
	ACR-A	90.5	87.5						
Gartsman and O’Connor [30]	ACR-SD	91.5							
	ACR	89.2							
Milano et al. [32]	ACR-SD				103.6			18.2	23.7
	ACR				96.1			23.1	26.2
Abrams et al. [29]	ACR	89.0		17.4	78.7	1.0	10.5		
	ACR-A	91.5		17.2	75.0	0.7	10.5		

There were no significant differences between the scores by procedure type. *ACR* Arthroscopic cuff repair, *ACR-A* arthroscopic cuff repair with acromioplasty, *ACR-SD* arthroscopic cuff repair with subacromial decompression, *ASES* American Shoulder and Elbow Surgeons score, *CM* Constant–Murley score, *DASH* Disabilities of the Arm, Shoulder, and Hand questionnaire, *SST* Simple Shoulder Test, *UCLA* University of California, Los Angeles score, *VAS* visual analog scale for pain, *WORC* Western Ontario Rotator Cuff Index, *Work-DASH* Work-Disabilities of the Arm, Shoulder, and Hand questionnaire

**Table 3:** Pooled analysis of ASES and Constant–Murley scores (frequency-weighted means)

Study	Scoring system	Mean (SD) score with acromioplasty	Mean (SD) score no acromioplasty	*P* value
Abrams et al. [29]; Gartsman and O’Connor [30]; MacDonald et al. [31]	ASES	30.0 (±7.0)	29.6 (±5.2)	0.938
Abrams et al. [29]; Milano et al. [32]	CM	44.5 (±2.0)	46.7 (±6.1)	0.673

*ASES* American Shoulder and Elbow Surgeons score, *CM* Constant-Murley score, *SD* standard deviation

## Discussion

Our systematic review of the literature showed no difference in short-term clinical results between patients with full-thickness rotator cuff tears who are treated with versus without acromioplasty and CA ligament release. Our results support the findings of the American Academy of Orthopaedic Surgeons [36], which gave acromioplasty a “moderate” recommendation for the treatment of rotator cuff disease. On the basis of two studies [30, 32] they suggested that “routine acromioplasty is not required at the time of rotator cuff repair,” and that despite theoretic benefits of acromioplasty in the setting of rotator cuff repair, it has little or no effect on postoperative clinical outcomes. Furthermore, one published systematic review and meta-analysis of three studies of patients undergoing arthroscopic rotator cuff repair treated with subacromial decompression found no difference from those treated without subacromial decompression [18].

There are several challenges when performing studies and interpreting the results of studies about rotator cuff disease. The first is the wide range of abnormalities that can be included under the umbrella of rotator cuff disease. The patient with “impingement” pain without any rotator cuff abnormality at the time of arthroscopy may be an entirely different entity from the patient who has a partial-thickness or full-thickness rotator cuff tear. Similarly, the degree of partial tear (in terms of percentage of depth of the tendon involved) may be a critical factor in determining the treatment [37]. The size of full-thickness rotator cuff tears has been shown to be a major factor in the success or failure of their treatment, and it is nearly impossible to have a study of the effect of treatment in patients with only one size of tear. Other abnormalities may also contribute to pain in this group of patients, such as biceps tendon abnormality or stiffness of the shoulder; these factors are rarely addressed in studies of the treatment of rotator cuff disease. Lastly, the origin of the pain in rotator cuff disease has not yet been established, making surgical treatment empirical.

There are other limitations of our study. There is wide variability in the reporting of results of surgery for rotator cuff disease. The results of any clinical study should include subjective patient measures (e.g., satisfaction, pain relief), patient- or disease-specific outcomes, preoperative versus postoperative range of motion, strength testing, and integrity of the rotator cuff repair at least 1–2 years after surgery. None of the studies reported here included all of these elements (Table 4). This variability makes it difficult to compare the results of all of the factors important to the surgeon and the patient. For example, in our systematic review, the variability in functional outcome measures reported across studies made a pooled analysis possible for only American Shoulder and Elbow Surgeons and Constant–Murley scores. Moreover, although this review included all RCTs reporting on outcomes after arthroscopic treatment of rotator cuff tears and/or “impingement syndrome,” the surgical techniques in the studies may have varied, creating the potential for performance bias. Lastly, the follow-up periods in the included studies ranged from 1 to 2 years. Larger studies with longer follow-up will be required to corroborate the reported findings regarding the need for partial acromioplasty with CA ligament release.

**Table 4:** Parameters evaluated in the included studies

Study	Pain relief	Patient satisfaction	Rotator cuff tear integrity	Shoulder strength testing	Patient- or disease-specific outcome measures
Abrams et al. [29]	Yes	No	No	Yes	Yes
Gartsman and O’Connor [30]	Yes	No	No	No	Yes
MacDonald et al. [31]	Yes	No	No	No	Yes
Milano et al. [32]	No	No	No	No	Yes

Although Neer [8, 9] remarked that acromioplasty should be reserved for “carefully selected patients with mechanical impingement” and proposed that this procedure should be performed only for patients with reasonable life expectancy and persistent disability despite at least 1 year of nonoperative treatment, Vitale et al. [38] showed that the incidence of acromioplasty has increased dramatically in recent decades. They analyzed the New York Statewide Planning and Research Cooperative System ambulatory surgery database from 1996 to 2006 and the American Board of Orthopaedic Surgery database from 1999 to 2008 to identify patients who had undergone acromioplasty. They reported a 254 % increase in the Statewide Planning and Research Cooperative System group versus a 142 % increase in the American Board of Orthopaedic Surgery group for the number of acromioplasties over their respective time periods. Yu et al. [39] also evaluated the rising incidence of anterior acromioplasty using medical records of residents in Olmsted County, Minnesota, who underwent isolated acromioplasty between 1980 and 2005. They found a 576 % increase over this time period, further showing the widespread popularity of this procedure. It is likely that because acromioplasty is no longer reimbursed by some insurers in the United States, the incidence of acromioplasty will begin to decrease.

Another issue that we were not able to address in this systematic review was the role of CA ligament release alone in the treatment of rotator cuff disease. Moorman et al. [40] performed a biomechanical study of the CA ligament and found that it was an important restraint to superior subluxation of the humeral head. They concluded that the CA ligament was not vestigial and served an important function in shoulder stability [40]. As a result, standard performance of the procedure has some theoretical disadvantages, including superior subluxation of the humeral head in some patients [40]. Unfortunately, there is no strong evidence for or against CA ligament release alone or in combination with other procedures for the treatment of the different stages and abnormalities of rotator cuff disease.

There is an increasing number of published reports examining the role of acromioplasty with concomitant CA ligament release in the treatment of rotator cuff disease. The current literature suggests that patients have similar outcomes at short-term and intermediate follow-up independent of whether acromioplasty was performed, regardless of acromion morphology. These findings do not support the routine use of acromioplasty as an adjunct to arthroscopic rotator cuff repair. However, current knowledge is limited by the unknown pathophysiology of rotator cuff disease and the inability to know exactly what produces a satisfactory result with rotator cuff surgery. Further study is needed to evaluate the role of acromioplasty and bursectomy alone in the treatment of rotator cuff disease.

## Appendix: Search strategy

### MEDLINE/PubMed

1. Acromioplasty/
2. Acromioplasty*.mp.
3. Exp acromioplasty/
4. Exp arthroscopic acromioplasty/
5. Exp open acromioplasty/
6. Exp subacromial decompression/
7. Exp coracoacromial ligament/
8. Arthroscopic acromiop*.mp.
9. Open acromiop*.mp.
10. Subacromial decomp*.mp.
11. Coracoacromial lig*.mp.
12. 1 or 2 or 3
13. 4 or 5 or 6 or 7 or 8 or 9 or 10 or 11
14. 12 and 13

### Excerpta Medica/EMBASE

1. Acromioplasty/
2. Acromioplasty*.mp.
3. Exp acromioplasty/
4. Exp arthroscopic acromioplasty/
5. Exp open acromioplasty/
6. Exp subacromial decompression/
7. Exp coracoacromial ligament/
8. Arthroscopic acromiop*.mp.
9. Open acromiop*.mp.
10. Subacromial decomp*.mp.
11. Coracoacromial lig*.mp.
12. 1 or 2 or 3
13. 4 or 5 or 6 or 7 or 8 or 9 or 10 or 11
14. 12 and 13

### Cochrane Register of Controlled Trials

A text-search strategy was performed using the terms “acromioplasty AND (arthroscopic* OR open* OR subacromial decompression* OR coracoacromial ligament*)”.
